# Toward Efficient Image Recognition in Sensor-Based IoT: A Weight Initialization Optimizing Method for CNN Based on RGB Influence Proportion

**DOI:** 10.3390/s20102866

**Published:** 2020-05-18

**Authors:** Zile Deng, Yuanlong Cao, Xinyu Zhou, Yugen Yi, Yirui Jiang, Ilsun You

**Affiliations:** 1 School of Software, Jiangxi Normal University, Nanchang 330022, China; chitoseyono@gmail.com (Z.D.); ylcao@jxnu.edu.cn (Y.C.); yg510@jxnu.edu.cn (Y.Y.); yiruijiang512@gmail.com (Y.J.); 2 School of Computer and Information Engineering, Jiangxi Normal University, Nanchang 330022, China; xyzhou@jxnu.edu.cn; 3 Department of Information Security Engineering, Soonchunhyang University, Asan 31538, Korea

**Keywords:** convolution neural network (CNN), image recognition, IoT application, *k*-nearest neighbor (*k*-NN)

## Abstract

As the Internet of Things (IoT) is predicted to deal with different problems based on big data, its applications have become increasingly dependent on visual data and deep learning technology, and it is a big challenge to find a suitable method for IoT systems to analyze image data. Traditional deep learning methods have never explicitly taken the color differences of data into account, but from the experience of human vision, colors play differently significant roles in recognizing things. This paper proposes a weight initialization method for deep learning in image recognition problems based on RGB influence proportion, aiming to improve the training process of the learning algorithms. In this paper, we try to extract the RGB proportion and utilize it in the weight initialization process. We conduct several experiments on different datasets to evaluate the effectiveness of our proposal, and it is proven to be effective on small datasets. In addition, as for the access to the RGB influence proportion, we also provide an expedient approach to get the early proportion for the following usage. We assume that the proposed method can be used for IoT sensors to securely analyze complex data in the future.

## 1. Introduction

The Internet of Things (IoT) is designed for making everything in our living environment integrated to improve quality of life [1]. With a wide range of sensor-equipped devices working together [2], the whole system is expected to accomplish a host of arrangements effectively with only minor human interactions [3]. In most cases, the data need to be obtained from visual sensing devices. For example, there is gesture recognition for unlocking in the smart home, image segmentation for judgments in autonomous driving, medical image analysis in the health care system, and so forth [4,5,6]. When an IoT system integrates with visual sensors like complementary metal oxide semiconductor (CMOS) image sensors [7], a huge amount of data will be collected or generated by them [8], and these data need to be analyzed fast in order to achieve better communication of devices in the IoT system [9,10].

While the mission to figure out hidden knowledge behind those complex data cannot be easily solved by the traditional methods, deep learning (DL) algorithms, an advanced branch of machine learning, are extremely necessary for IoT applications [11]. The aforementioned smart home, self-driving, and smart health systems are all prime examples. Computer vision with DL is indispensable for the whole system to ensure its functions [12,13]. In recent years, IoT researchers have been faced with problems when trying find a more appropriate way to implement CV and DL technologies. They came up with various approaches to improve machine learning algorithms and design frameworks for faster data analytics [14,15], but how to speed up the analysis process much further remains a important problem.

To address the aforementioned problem, this paper proposes an improved method for initialization in DL for image recognition problems based on the RGB influence proportion. The method is designed to take the influence of RGB information out of the data from visual sensors into consideration for the training process. The proposed method is based on a phenomenon observed from human beings that people catch different colors from each of their eyeballs, and each of them plays a different role for perception [16]. Even though the idea is already included in the convolution layers in CNN, it has not been tried on the initialization process. With the influential proportion of RGB channels, we can modify the initialization approach for image processing problems so as to make it come into effect during the training phase. To test the proposed method, different sizes of datasets and learning models, including a convolution neural network (CNN) and a fully connected neural network are configured to figure out the effectiveness of RGB-based initialization for image recognition jobs. As the effect of the initialization method needs to be observed in the training process, the learning curves are recorded to evaluate the performance. Additionally, a convenient way with *k*-nearest neighbor (*k*-NN) to get the early RGB proportion is provided and tested in our work. To be more precise, this proposal has made the following major contributions:

(i) It presents an initialization option for CNN to make use of RGB proportions, which can be modified in the future, and thereby shortens the convergence of the learning models.
(ii) It proved that *k*-NN can be used to extract the early proportion for the RGB-based initialization, which can be deemed as a pre-training process.

The remainder of this paper is organized as follows. Related work of our paper is described in Section 2. Section 3 mentions the proposed work as well as some details of our experiments, and the corresponding results with their analysis are presented in Section 4. Section 5 recommends some future work and concludes the paper.

## 2. Related Work

### 2.1. Weight Initialization Methods

With the special nature of multilayer architectures, deep learning methods are able to reinforce different fields and finish more complicated missions, which makes them enjoy a high reputation in the field of machine learning [17]. As a typical nonlinear method, it shows great performance in training flexibility and dealing with the complicated nonlinear relationship in data [18]. Nowadays, after improvements in various dimensions, deep learning shows outstanding success in different areas, including natural language processing (NLP) [19], speech recognition [20], computer vision [21], etc.

However, being a nonlinear method also means that DL algorithms suffer from high variance. In such cases, neural networks are very sensitive to some initial conditions, such as the size of the dataset and the initial weights, in particular, which play an essential part in neural networks [22]. A good weight initialization aims at helping the neural network to preserve a stable learning tendency and to shorten the convergence time [23]. Without a suitable weight initialization, the network may be trained at the deadly risk of creating an exploding gradient or vanishing gradient, which can result in a very slow convergence or even an inability to converge. Thus, it is of great importance to choose a proper weight initialization approach when training a network [24].

Zero initialization is never a viable approach, as it makes the gradient of every layer the same value during back propagation. Random initialization performs better than the zero one, but it is not easy to find the appropriate range of random values because if weights are initialized too high or too low, problems like gradient vanishing or gradient explosion can still cause errors during training [25]. Recently, a variety of weight initialization approaches were put forward to cope with this problem, including Gaussian distribution initialization.

As is shown in Table 1, Xavier et al. aimed at keeping the variances of input and output data the same to ensure that zero output phenomenon can be avoided, but their method is only based on linear functions [26]. To deal with this, He et al. made some modifications to Xavier’s method [27]. After those improvements, the initialization could well perform when using ReLU as the activation function.

These methods have been proven to be expedient in weight initialization in neural networks and are now very popular in the deep learning field [28]. However, in computer vision, data with RGB channels are usually encountered. That is because, in addition to texture, shape, and material, color also plays an important part in recognizing things for human beings [29]. To mimic human vision, RGB color mode was designed, and data on computers now are finally filled with colors. Colors are the basic elements in vision problems, as they can be treated as detailed data contributing to greater performance of image recognition [30], object detection [31], etc. Additionally, when humans deal with colors caught by the different cone cells in the retina (red, green, and blue cells), the perception differs according to the degree of each color—named the RGB influence proportion herein.

Traditionally, the channels are regarded as the third dimension of data input in most of the CNN-based models [32]. However, under different datasets, the RGB influence proportion varies, which means that there can be a specific channel or two channels more influential than the others. If we can utilize the RGB influence proportions to modify the random weights at the beginning, the whole model might be prone to being influenced more by those most influential channels, so that the model might converge faster [33]. Thus, we suppose that we can improve the weight initialization method in image recognition based on the RGB influence proportion.

### 2.2. K-Nearest Neighbors Algorithm

*k*-NN is a simple but effective machine learning algorithm. Based on the distances from the training sample, *k*-NN needs no training process for the result, making it the representative of “lazy learning” [34]. *k*-NN finishes a prediction by referring to the majority vote from *k* nearest samples from the input object in the feature space [35].

It is necessary to get the proportion before implementing RGB-based weight initialization. Since the RGB influence proportion is not always a provided value, an approach to gaining an early proportion is in great need. In the meanwhile, as the *k*-NN algorithm enjoys a great reputation for its prediction speed and simple theory [36], we can use *k*-NN to get the early RGB proportion for the subsequent initialization. Even though the accuracy is not great, but the proportion of accuracy can be used as the early one for RGB-based initialization.

Since it is not our target to get great accuracy in this stage, and also the process of *k*-NN is not drawn out, we perform a small grid search over the number of neighbors *k* from 1 to 30 to observe the most appropriate *k* for extrapolating the RGB proportion.

## 3. Materials and Method

**Figure 1:**
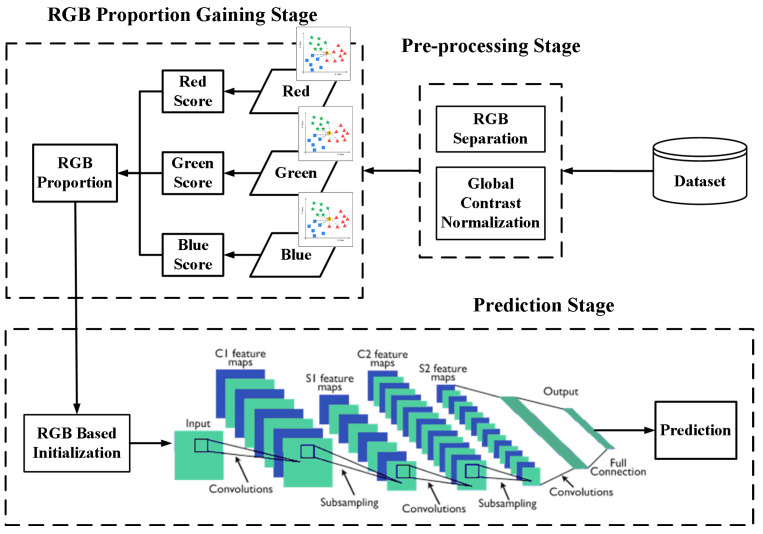
Basic framework of the proposed RGB-based initialization.

The basic framework of our proposed method is illustrated in Figure 1. Generally, the whole process can be divided into two phases: gaining the proportion and using it in the initialization phase. In order to utilize the RGB influence proportion, we need to obtain it first. Despite the pre-processing stage for cleaning data from the visual sensors, those input data need to run through an RGB proportion gaining stage to obtain the early proportion, which is going to be applied to the weight distribution. After that, those data will be treated as an input for DL models, such as CNN, and the training process, and the final accuracy will be recorded for evaluation of this initialization method.

### 3.1. Utilizing the RGB Influence Proportion

**Figure 2:**
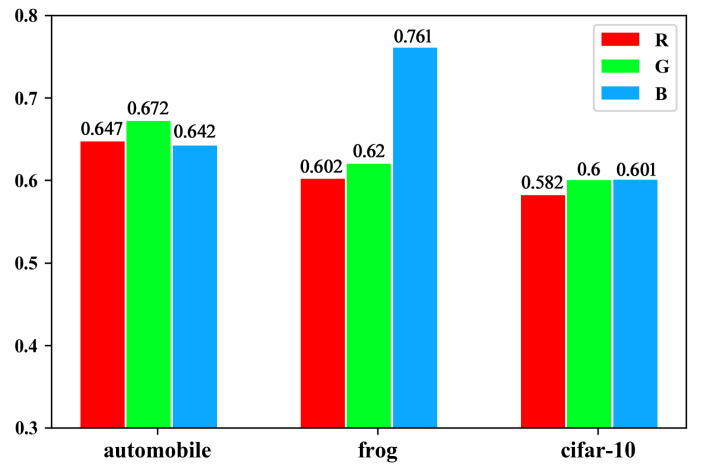
RGB influence proportion in some dataset within CIFAR-10.

In our previous work [37], to find out the different RGB influences on image recognition problems, we designed experiments that trained three CNNs on 3 different datasets separated by RGB channels from one dataset. The results showed that in terms of different object classifications, the performance of each CNN varies. In other words, RGB channels show, respectively, different influence proportions. As is shown in Figure 2, the three channels have different significance levels; e.g., the frog classification, where the RGB influence proportion is 0.91:0.94:1.15 (all numbers of proportions are transformed; e.g., 0.602:0.62:0.761≈0.91:0.94:1.15 here). Some of them show slight differences, such as automobile classification—0.99:1.04:0.99. Additionally, we obtain the RGB proportion of the whole dataset, which is 0.98:1.01:1.01. We suppose that we can improve the weight initialization based on the influence proportion of RGB channels when the proportion is appreciable enough. It seems that the effectiveness will be less significant when it comes to some large datasets whose influences on the differences of RGB channels are not obvious, including some datasets full of colors and some comprehensive datasets, but have conducted experiments on them to find out the real effect.

To verify our idea above, we design different experiments, one of which is to find out the effectiveness of RGB-based initialization on small datasets containing frog and automobile images split from CIFAR-10 dataset, and another one is conducted on the whole dataset. For the reason that the difference of RGB influence in frogs is more appreciable than the automobile one, we stack them up to build a small dataset that relatively has a difference of RGB influence (R:G:B=0.96:0.98:1.06). The output layers of different CNNs are modified a little according to their classification jobs, but their basic structures are the same as a whole. Additionally, we conduct the experiment on a fully-connected network in a small dataset to find out whether it is practicable in a fully connected neural network.

As for how to utilize the color differences, we assume that we can generate the weight distributions according to their influence proportions. In this case, no matter what kind of neural network we are using, the RGB-based initialization is only applied in the first layer, as it is directly connected to the input data. However, there are little differences in initialization methods for different learning models because of their different mechanisms.

**Figure 3:**
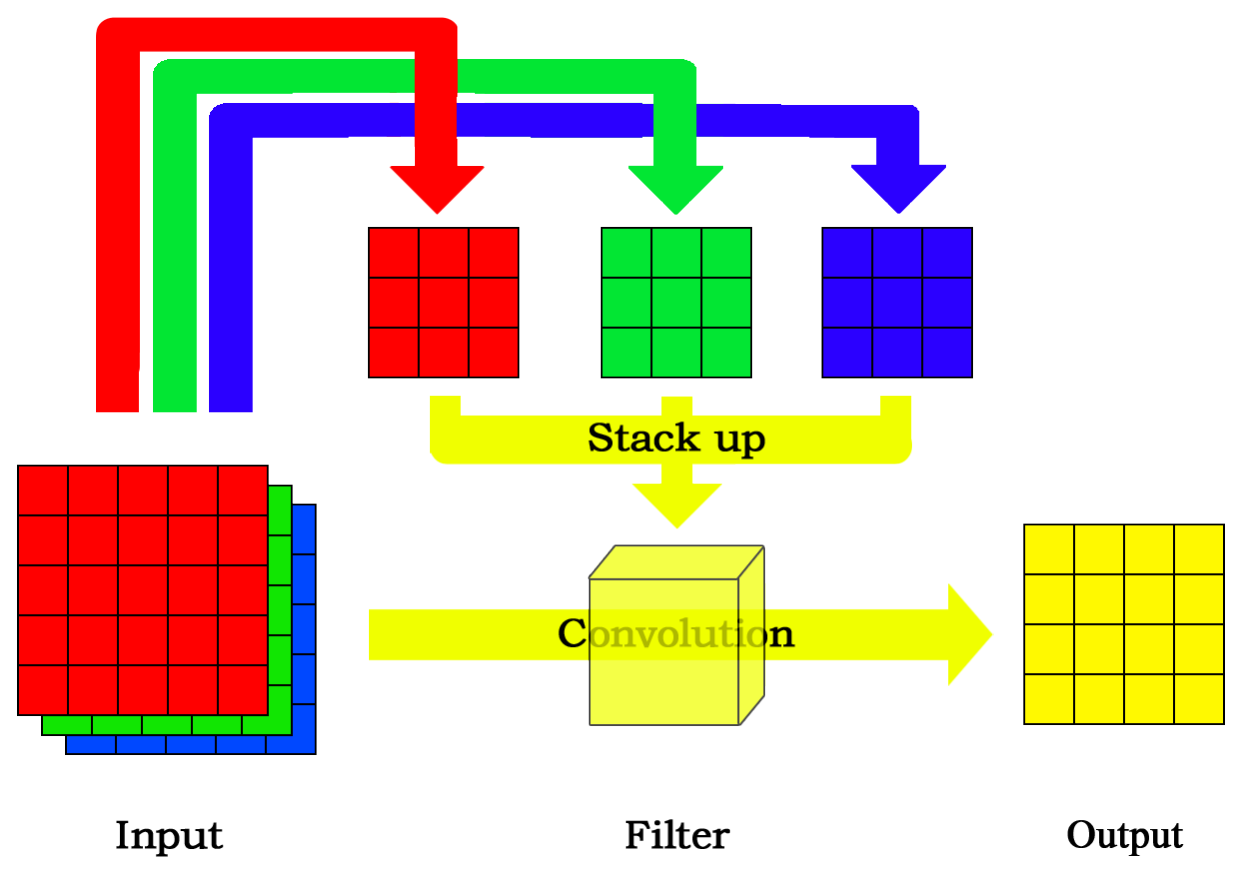
RGB initialization implementation in convolution neural network (CNN).

In CNN, we first get the RGB influence proportion from the dataset by other learning methods and then generate the random distributions for weights of different channels according to the obtained proportion. As there are three channels, we can get three different kernels distributed to three different distributions. Then we stack them up as one convolution filter for the first layer. Figure 3 shows that we set the weight proportion of each channel of the kernels according to the RGB proportion and the process of stacking up into a whole filter.

**Figure 4:**
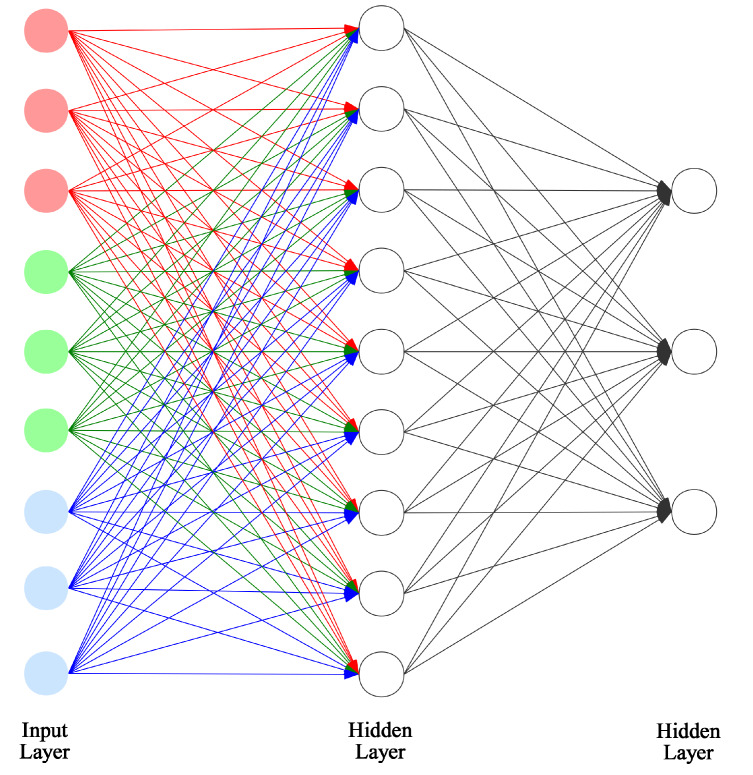
RGB initialization implementation in a fully-connected network.

In a fully connected neural network, there are not any structures like filters. As is shown in Figure 4, we have to divide the neurons of input layers into three parts equally. In this case, because in the first layer, each part deals with its only channel, we can group all of them by their corresponding channels, and set the weight distribution of each part according to the RGB proportion just like what we did for each kernel in CNN.

### 3.2. Experiment Configurations

#### Learning Models

In this section, we describe the baseline framework of CNN we use throughout the corresponding experiments. Table 2 illustrates the overall framework of our experiments.

Every experiment here shares almost the same architecture except for the output layer. During the training stage, the input to our networks is a 32×32 RGB image. We only apply a 0–1 normalization in the pre-processing stage by dividing every datum by 255. All convolutional layers are filled with filters with the size of 3×3 and rectification non-linearity (ReLU) [38] as the activation function. Besides, we apply the same padding method in filters to capture every pixel of the images. We also attach several max-pooling layers after some of the convolution layers (some convolution layers are followed by dropout layers with a ratio of 0.3) [39], which are performed on 2×2 windows, with stride 2.

After the convolution stage, there are three fully connected layers, whose activation functions are also ReLU except for the output layer. In different experiments, the output sizes of soft-max function in the final layers differ (two neurons on the small dataset and 10 on the whole dataset).

As we also conduct the experiment on a fully-connected network; its overall structure is demonstrated in Table 3. The total structure is pretty simple. With the same input layer as the CNN, all we have here are fully connected layers followed by dropout layers.

### 3.3. Weight Initialization

**Figure 5:**
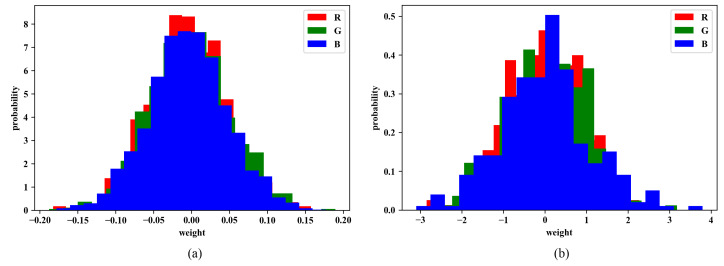
Examples of weight distribution (**a**) after normal initialization, and (**b**) after RGB-based initialization.

As for the most important part in our experiments, weight initialization, we apply different approaches for comparison. We separately used normal distribution and the RGB-based one. Initializing weights randomly from a normal distribution is the most common method for weight initialization. The figures of weights *w* are distributed to the standard normal distribution, with mean = 0 and standard deviation = 1. Figure 5a shows an example from this distribution.

Then, the RGB-based initialization we use here is based on the standard normal distribution. Given the influence proportion of RGB channels is separately $P_{r}$,$P_{g}$,$P_{b}$, we have the weight distribution of the red channel as below:

(1) $$P\left(w\right)=\frac{P_{r}}{\sqrt{2\pi }}\exp^{\left(-\frac{w^{2}}{2}\right)}.$$

Those of other channels are in the similar way. As for their proportion, take the proportion of red channel $P_{r}$ as an example again; we have

(2) $$P_{r}=\frac{A_{r}}{A_{r}+A_{g}+A_{b}},$$

where $A_{r},A_{g},A_{b}$ respectively represent the accuracy from the early prediction. Additionally, an example of this distribution is shown in Figure 5b with proportion $P_{r}:P_{g}:P_{b}=0.96:0.98:1.06$ of the small dataset we stacked up above.

### 3.4. k-NN for the Early Proportion

As one of the most widely used algorithms in pattern recognition, *k*-NN is easy to implement. In *k*-NN, *k* denotes the number of nearest neighbors that we choose to give the prediction. The equations in this section refer to T. Cover et al’s work [36].

To calculate the distance, Euclidean distance is most popularly used. The standard Euclidean distance is defined below.

(3) $$d(x_{i},x_{0})=\sqrt{\sum_{j=1}^{n}{\left(x_{i}^{\left(j\right)}-x_{0}^{\left(j\right)}\right)}^{2}},\;$$

where one of the *k* nearest samples from training dataset $x_{i}$ representing the feature vector of one sample $x_{i}$ = ($x_{i}^{\left(0\right)}$,$x_{i}^{\left(1\right)}$, …,$x_{i}^{\left(n\right)}$) and $x_{0}$ from the testing dataset have the same size.

Then, it computes the most common class in its *k* nearest neighbors to predict its class. The algorithm is given below

(4) $$\hat{f}\left(x_{0}\right)=\underset{v\in V}{\arg\min}\sum_{j=1}^{k}\delta (v,f\left(x_{i}\right)),\;$$

where *V* = $v_{1}$, $v_{2}$,..., $v_{s}$ represents the discrete labels, and also $f:R^{n}\to V$ projects the corresponding label of $x_{i}$.

Besides, we adjust the number of *k* from 1 to 30 to see the most appropriate result. For the reason that the result we need from *k*-NN is barely the influence proportion of RGB channels instead of an impeccable accuracy, we just need to find a certain *k* when their accuracies show the biggest differences.

## 4. Performing Experiments

### 4.1. Experiment Setup

In this section, we perform all the experiments described above using Keras 2.2.4 (François Chollet, Mountain View, CA, USA) with Tensorflow 1.13 (Google Brain, Mountain View, CA, USA) as its backend [40,41]. As we assumed that the effect of RGB-based initialization probably has some differences in datasets with different sizes, we perform comparison experiments to observe the difference and also propose a method to obtain the early weight proportion. All experiments are based on the CIFAR-10 dataset [42]. In our experiments, the frog and automobile images (12,000 totally, 8000 for training, 2000 for validation, 2000 for testing) from the CIFAR-10 training dataset and testing dataset build up the small dataset. Additionally, accuracies from the training dataset (40,000 images) with the validation dataset (10,000 images) split from the CIFAR-10 training dataset (50,000 images) are presented for comparison.

We carry out the training process by optimizing the cross-entropy using mini-batch gradient descent with adaptive moment estimation (Adam) [43]. We set the batch size as 128 and train the models respectively for 50 epochs and 100 epochs under different datasets (50 for small one and 100 for the whole one).

As we mentioned above, the same experiment is conducted on a fully-connected network to see differences. Since it is proven that under the whole dataset, the proposed method is not useful as it under the small one, we only carry out the experiment for a fully-connected network under the small one this time.

As for the configuration for the *k*-NN experiment, we conduct the experiment on the same dataset as the small one split from the whole CIFAR-10 in CNN. By separating the dataset into three datasets, we train three *k*-NNs to sort out the influences of different RGB channels. In this case, their accuracy can be deemed as their early proportion. Additionally, a grid search is applied to gain the most proper *k*.

### 4.2. Experimental Results

#### 4.2.1. Results of Proposed Method on CNN

**Figure 6:**
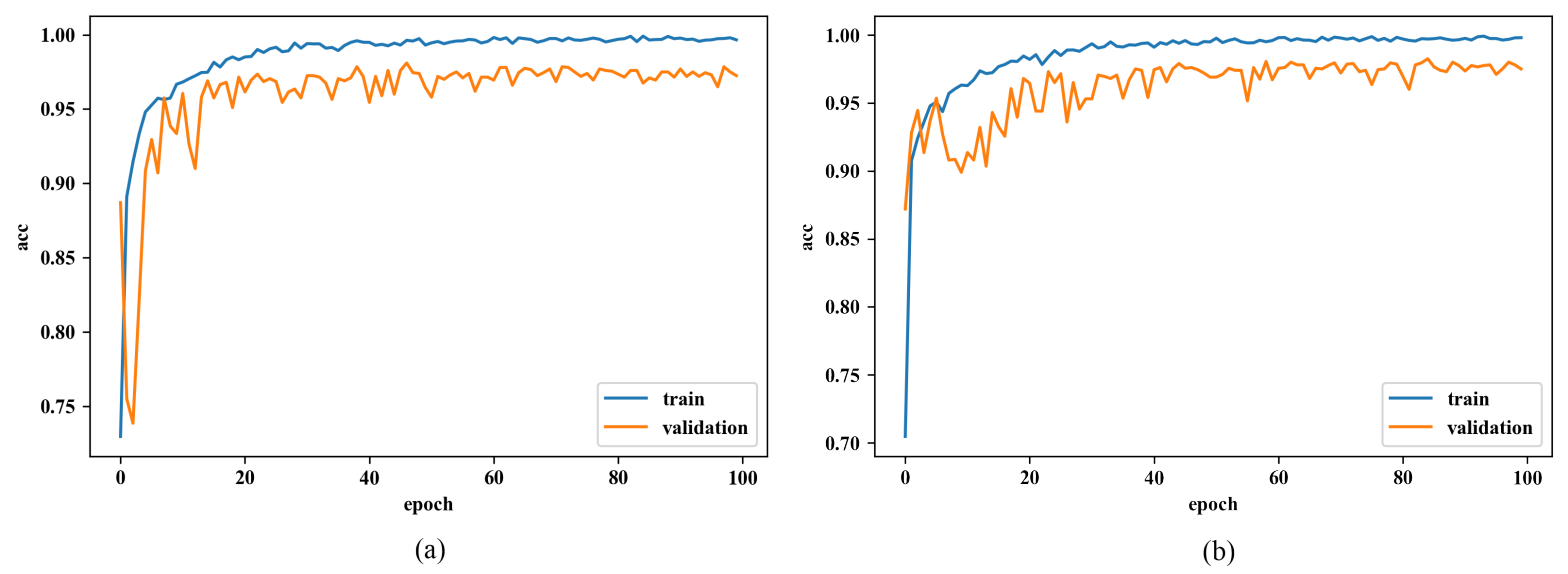
Learning curve of CNN under the small dataset (**a**) using normal initialization, and (**b**) using RGB-based initialization.

**Figure 7:**
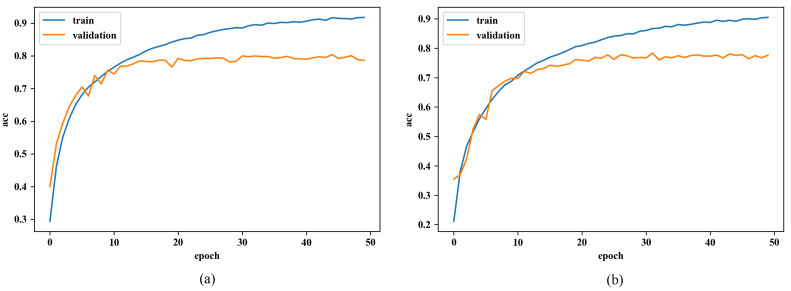
Learning curve under the whole dataset (**a**) using normal initialization, and (**b**) using RGB-based initialization.

We begin with the performance evaluation on a small dataset; the whole dataset was used. The comparison of learning curves using different initialization methods and under different datasets are respectively shown in Figure 6 and Figure 7.

Firstly, in the case of the small dataset, it is notable that even though both accuracies from the training dataset (blue curve) show similar increasing trends, the figure from the RGB-based one is steeper than the other one. Additionally, the distinction of validation accuracy between both networks (orange curve) is quite appreciable, such that accuracy of the RGB-based one is already close to the accuracy of convergence at the very beginning, while the normal one takes epochs to reach that value. It shows that the RGB-based initialization somehow contributes to faster convergence on CNN. But after several epochs at the beginning, the accuracy from the RGB-based one underwent a drop and rose again. Secondly, the validation accuracies of both networks fluctuate in the range of approximately 0.90–0.98 after first increasing to more than 0.90. After the training process, they gain an identical score on the test dataset. Namely, the RGB-based initialization has no influence on the performance after convergence.

In addition, we also conduct the same experiment on the whole dataset to see whether the initialization approach is suitable for a large dataset. However, in spite of some nuances, the performance of the network with or without the RGB initialization sees a negligible difference. The trends are the same and so are the prediction scores after training.

To sum up, after evaluating the performance of our initialization on CNNs under the dataset of different sizes, we know that, to some extent, the RGB-based initialization approach does have a positive influence on convergence during training when the proportion is appreciable, but it is only useful in the first 5–7 epochs, and after the same process of training, models seem the same with or without this initialization. Additionally, it turns to be insignificant in the whole dataset, since the dataset consists of many types of images when the RGB channels show respectively similar influences on image recognition (R:G:B=0.98:1.01:1.01).

#### 4.2.2. RGB-Based Initialization on a Fully-Connected Network

**Figure 8:**
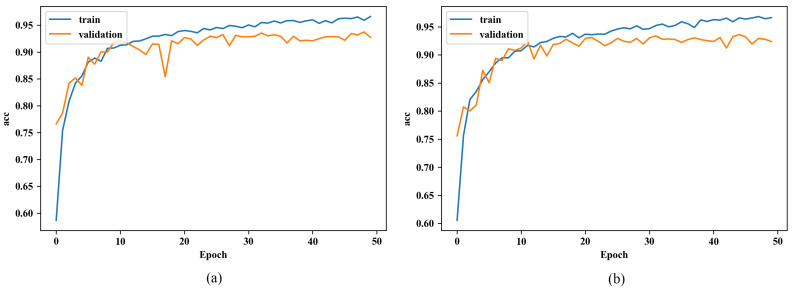
Learning curve of fully connected neural network (**a**) using normal initialization, and (**b**) using RGB-based initialization.

From the Figure 8, we can see that both curves are almost identical. Their validation accuracies both converge to 0.92 and their train accuracies show totally the same trend. It is out of expectation that the performances with different initialization are almost the same. We can tell that this RGB-based method is not applicable for fully-connected networks.

#### 4.2.3. Evaluating the Changes of Weights

**Figure 9:**
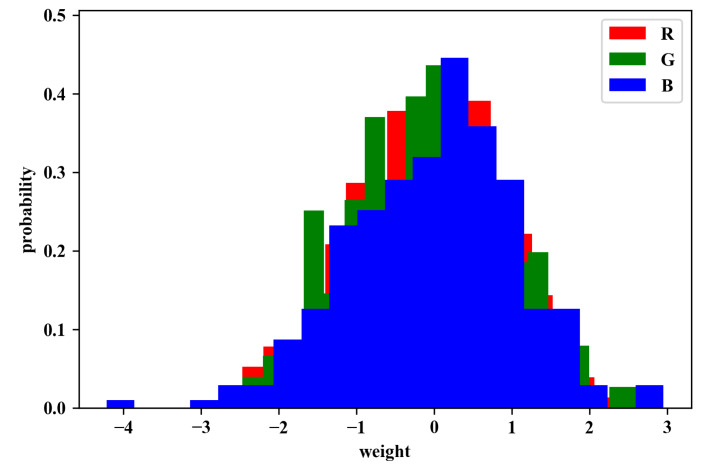
Example of weight distribution after CNN trained.

After Experiment 1, we perform an additional experiment to record the weight distribution in order to further prove the effectiveness of our initialization. These weights are extracted from the first layer of CNN, which is also the weights we did modifications on at first. As Figure 5 shows the weight distribution from RGB channels in the normal distribution and the one in the RGB-based distribution before training; it is clear that distributions from different channels are similar in the normal distribution, while in RGB-based one, the blue channel is apparently wider and higher than others. Figure 9 illustrates the distributions after training, in which the distribution of the blue channel is the widest and highest one, which is similar to the one just after RGB-based initialization. It also shows the faster convergence might be because the initial weights are already close to the convergence values at the beginning.

#### 4.2.4. Early Proportion by *k*-NN

**Figure 10:**
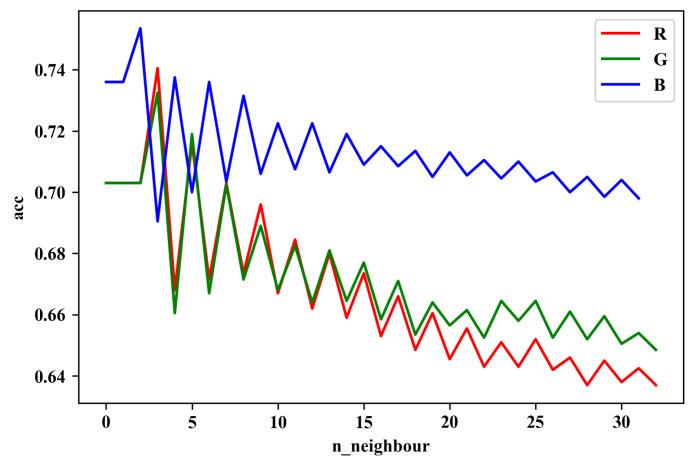
Results of *k*-NN on different k between 1 and 30.

The result is shown in Figure 10, where it is clear to see that *k*-NN trained on the blue dataset always has the best performance. What is more, as the number *k* increased, the accuracies among three *k*-NN decreased, while interestingly, the difference between green and red dataset increasingly widened, and was similar to the origin proportion in that green was marginally larger than red.

Then we pick their accuracies when *k* = 25, where their distinction is seemingly more significant. The accuracies of different datasets are respectively 0.65, 0.67, and 0.71. Thus, the early proportion we got here is 0.96:0.99:1.04, which is quite similar to the one we detect by CNN that 0.96:0.98:1.06. Therefore, we can tell that *k*-NN is useful to get the approximate early RGB proportion for initialization.

## 5. Discussion

From the experimental results, we can see that the effectiveness of the proposed RGB-based initialization is limited compared to the traditional initialization methods (only influences in the first few epochs). However, it is worth mentioning that the proposed method is the first study to apply pre-training to extract color differences and utilize it in the initialization process. We here discuss the limitations of our proposal and highlight some interesting problems. We hope to attract more researchers to notice this topic and push the boundaries forward.

(i) As far as a specific dataset is concerned, the RGB influence proportion is existed and fixed. It is not a variable. It is supposed to be given by some other means. In this paper, we got the proportion by ensemble learning and *k*-NN, while one is too intricate and another one is slightly inaccurate. A more convenient way to compute the proportion is still in need. Besides, if the RGB proportion can be made as a property of the dataset, whoever needs to use the proportion can make use of it directly without measuring the proportion by himself.
(ii) The approach we use to deal with the RGB proportion is quite simple right now, as we just apply it on the original standard normal distribution and utilize it on the first layer of the model, even though it is proven to be useful in first epochs. Firstly, the not ideal result might be the consequence of using a learning model with too many layers, so that the total influence is reduced. Additionally, we can try forward propagation after generating the first layer to fully use the color difference. These are both worth further investigation and experimentation.
(iii) The difference among three channels from the dataset we used here is not very significant in this work. Thus, a more specific dataset which includes data with higher RGB proportions is recommended. Additionally, it is worth mentioning that in some image recognition cases, color difference might be much more evident. In that case, the proposed method will be more effective.
(iv) Currently, there are different initialization methods, such as He and Xavier initialization, which can also be applied like the proposed method but probably with better performance. A controlled experiment with them is recommended so that the conclusion can be more trustworthy.
(v) Recently, it has been a great trend of the IoT system to include more visual sensor-based devices and keep data security in the process of analysis. In order to realize it, the proposed method which is used to optimize the training process can be utilized in IoT systems after some modifications and improvements in the future.

## 6. Conclusions and Future Work

Motivated by the fact that color difference plays an important part in image recognition, this paper proposed a novel solution to explicitly make use of the RGB proportion in the initialization process for CNN. The proposed method was applied based on the traditional initialization method, which is designed to use a pre-training method to emulate the RGB distribution after being trained. We carried out several experiments not only find out the effectiveness of the RGB-based weight initialization, but also to come up with a useful method to implement the initialization in order to speed up training processes. This proposed method (i) can be utilized to optimize the convergence performance in the cases in which the color influence difference of the dataset is obvious, (ii) and propose a possible direction to drive the research about pre-training forward. (iii) Additionally, we proved that *k*-NN can be used to compute the early proportion for initialization. Since we illustrated that the RGB-based-initializing scheme is not valid in all cases when the color influence differences are not obvious, the early proportion from *k*-NN can help to recognize whether the proposed method is useful or not.

Although making use of RGB proportion in generating the weight distribution the first layers is proven to be somehow useful, there is some important future work that needs to be done to push it further. Currently, our future work will try to utilize the color influence difference in the whole network—the most important task for our future work—to improve performance. Furthermore, how to apply the proposed method in other advanced methods such as Xavier and He initialization is an important task, and the comparison with different base-initialization methods will be presented to further prove the effectiveness of the proposed method.

## Figures and Tables

**Table 1:** Research gaps in the related work.

Method	Using Color Influence Differences	Influence on Convergence
Traditional CNN	In the convolution process	/
Gaussian distribution initialization	/	/
Xavier Initialization	/	By keeping variance consistent
He Initialization	/	Xavier’s method based on ReLU

**Table 2:** CNN model configuration for the experiments.

Layer (Type)	Output Shape	Param
input (32×32 RGB image)		
conv2d_1 (Conv2D)	(None, 32, 32, 32)	896
dropout_1 (Dropout)	(None, 32, 32, 32)	0
conv2d_2 (Conv2D)	(None, 32, 32, 32)	9248
max_pooling2d_1 (MaxPooling2)	(None, 16, 16, 32)	0
conv2d_3 (Conv2D)	(None, 16, 16, 64)	18,496
dropout_2 (Dropout)	(None, 16, 16, 64)	0
conv2d_4 (Conv2D)	(None, 16, 16, 64)	36,928
max_pooling2d_2 (MaxPooling2)	(None, 8, 8, 64)	0
conv2d_5 (Conv2D)	(None, 8, 8, 128)	73,856
dropout_3 (Dropout)	(None, 8, 8, 128)	0
conv2d_6 (Conv2D)	(None, 8, 8, 128)	147,584
max_pooling2d_3 (MaxPooling2)	(None, 4, 4, 128)	0
flatten_1 (Flatten)	(None, 2048)	0
dense_1 (Dense)	(None, 2500)	5,122,500
dropout_5 (Dropout)	(None, 2500)	0
dense_2 (Dense)	(None, 1500)	3,751,500
dropout_6 (Dropout)	(None, 1500)	0
dense_3 (Dense)	(None, 10)	15,010

**Table 3:** Fully connected model configuration.

Layer (Type)	Output Shape	Param
input (32×32 RGB image)		
dense_1 (Dense)	(None, 10,000)	30,730,000
dropout_1 (Dropout)	(None, 10,000)	0
dense_2 (Dense)	(None, 1000)	10,001,000
dropout_2 (Dropout)	(None, 1000)	0
dense_3 (Dense)	(None, 100)	100,100
dropout_3 (Dropout)	(None, 100)	0
dense_4 (Dense)	(None, 2)	202
